# Increased expression of A Proliferation-inducing Ligand (APRIL) in lung leukocytes and alveolar epithelial cells in COPD patients with non small cell lung cancer: a possible link between COPD and lung cancer?

**DOI:** 10.1186/s40248-016-0051-6

**Published:** 2016-04-04

**Authors:** Francesca Polverino, Maria Laucho-Contreras, Joselyn Rojas Quintero, Miguel Divo, Victor Pinto-Plata, Lynette Sholl, Juan P. de-Torres, Bartolome R. Celli, Caroline A. Owen

**Affiliations:** Division of Pulmonary and Critical Care Medicine, Brigham and Women’s Hospital and Harvard Medical School, Room 855B, Harvard Institutes of Medicine Building, 77 Avenue Louis Pasteur, Boston, MA 02115 USA; Lovelace Respiratory Research Institute, Albuquerque, NM USA; University of Parma, Parma, Italy; Department of Pathology, Brigham and Women’s Hospital and Harvard Medical School, Boston, MA USA; Clinica Universidad de Navarra, Pamplona, Spain

**Keywords:** Adaptive immunity, APRIL, Autoimmunity, COPD, Innate immunity, Non small cell lung cancer

## Abstract

**Background:**

Chronic Obstructive Pulmonary Disease (COPD) is characterized by an excessive activation of the adaptive immune system and, in particular, uncontrolled expansion of the B-cell pool. One of the key promoters of B cell expansion is A PRoliferation-Inducing Ligand (APRIL). APRIL has been strongly linked to non small cell lung cancer (NSCLC) onset and progression previously. However, little is known about the expression of APRIL in the lungs of COPD patients.

**Methods:**

Using immuno-fluorescence staining, the expression of APRIL was assessed in sections of lungs from 4 subjects with primary diagnosis of COPD (FEV_1_ 33 ± 20 % predicted), 4 subjects with primary diagnosis of NSCLC, 4 subjects diagnosed with both COPD and NSCLC, smokers without COPD or NSCLC and 3 healthy never-smokers. The percentage of B cells, alveolar macrophages (AMs) and polymorphonuclear neutrophils (PMNs) in the lung and alveolar epithelial cells (AECs) that stained positively for APRIL was quantified using epi-fluorescence microscopy and image analysis software.

**Results:**

The percentage of APRIL-expressing B cells, AMs, PMNs and alveolar epithelial cells (AECs) was higher in patients having both COPD and NSCLC than in patients with either COPD or NSCLC alone, SC or NSC (*p* < 0.03 for all comparisons). The percentage of APRIL-expressing AMs and AECs (but not in B cells) was higher in patients with NSCLC alone than in patients with COPD alone. The percentage of APRIL-expressing AECs (but not B cells or AMs) was higher in COPD patients than in SC and NSC (*p* < 0.05 for all comparisons). The percentage of APRIL-expressing B cells, AMs and AECs cells was similar in NSC and SC.

**Conclusion:**

The percentage of APRIL-expressing B cells, AMs and AECs is higher in the lungs of patients with both COPD and NSCLC than in patients with COPD or NSCLC alone or control subjects. These findings suggest that APRIL may contribute to the pathogenesis of both COPD and NSCLC, and possibly to the development of NSCLC in patients with established COPD.

## Background

Chronic obstructive pulmonary disease (COPD) is characterized by an enhanced pulmonary and systemic inflammatory response to inhaled particles and gases, particularly those found in tobacco smoke. COPD patients have an increased risk of developing non-small cell lung cancer (NSCLC) that is independent of smoking pack-year history [1, 2]. However, the factors that promote the development of NSCLC in COPD patients are not clear. Reactive oxygen and nitrogen species (ROS and RNS) derived from both exogenous and endogenous sources drive many of the pathways in both COPD and lung cancer [3]. ROS and RNS can react directly with DNA causing DNA damage, or impair DNA repair processes [4]. Increased lung oxidative stress levels can also increase the susceptibility of COPD patients to recurrent respiratory tract infections, and drive chronic inflammation in the lungs, leading to further DNA damage and cellular injury by inducing the production of cytokines and proteinases in the lung [5]. Injury to the lungs that is induced by chronic inflammation triggers repair processes including cellular proliferation which, together with ROS and RNS-induced DNA damage, may promote tumorigenesis.

Both innate immune cells (such as PMNs and alveolar macrophages [AMs]) and adaptive immune cells (T and B lymphocytes) participate in the chronic inflammatory responses occurring in COPD lungs [6–10]. This process does not resolve after cessation of smoking, suggesting that self-perpetuating mechanism(s) (similar to those occurring in autoimmune disease [8, 11]) are involved. This autoimmune response likely involves activation of (auto reactive) B cells [12] as the number of B cells in small airways is increased in severe and very severe COPD [13]. In contrast to COPD, there is a negative correlation between the severity of T lymphocyte infiltration in the lung and NSCLC disease progression [14–16]. As well as in COPD patients, NSCLC patients have tertiary lymphoid structures (TLS) characterized by clusters of mature dendritic cells and T cells surrounded by B cells, and all stages of B-cell differentiation are detectable in most NSCLC tumors [17]. A high density of follicular B cells correlates with long-term survival, both in patients with early-stage NSCLC and with advanced-stage NSCLC treated with chemotherapy [17]. However, IL-10-producing immunosuppressive regulatory B cells are also increased in NSCLC, and their numbers correlate directly with disease progression [18]. Thus, the roles of B cells (and their subsets) in regulating the development of NSCLC remains unclear.

Regulation of B-cell homeostasis involves two members of the TNF-alpha family: B-cell Activating Factor (BAFF) and A proliferation-inducing ligand (APRIL, TNFSF13, TRDL-1, *T*NF-*r*elated *d*eath *l*igand-*1*), which share two receptors (B-cell maturation antigen (BCMA) and transmembrane activator and calcium-modulator and cyclophilin ligand interactor (TACI). A third receptor, the BAFF receptor, only binds to BAFF [19]. BAFF and APRIL are key regulators of the immune response and are produced by myeloid leukocytes, lymphocytes and epithelial cells [20–25]. BAFF and APRIL both promote peripheral B-cell survival, maturation, and differentiation and play important roles in the production of antibodies. Serum levels of BAFF and APRIL are increased in several autoimmune diseases [26, 27].

APRIL is a type II membrane protein and a soluble form is produced by several cells [28]. APRIL expression is upregulated at the transcript and protein levels in NSCLC tumor cells and stromal fibroblasts [29, 30]. High-level expression of ARRIL in stromal cells was associated with impaired differentiation of stromal cells, and APRIL expression levels in tumor cells are an independent prognostic factor for 5-year survival in patients with NSCLC [30]. While some studies report that APRIL promotes the growth of some tumor cells [31], other studies report that APRIL promotes apoptosis of other tumor cell types [32, 33]. However, nothing is known about the expression of APRIL in immune cells or lung epithelial cells in NSCLC patients. Also, it is not clear whether the expression of APRIL is altered in the lungs of COPD patients or patients having both COPD and NSCLC. Herein, we sought to address these knowledge gaps by measuring APRIL expression in lung sections from patients with primary diagnosis of COPD or NSCLC, patients with both COPD and NSCLC, smokers without lung disease and never-smokers. We also identified the leukocyte subsets and lung epithelial cell types that express this molecule in these diseases.

## Methods

### Subjects and ethics

#### Lung tissue cohort characteristics

**Fig. 1:**
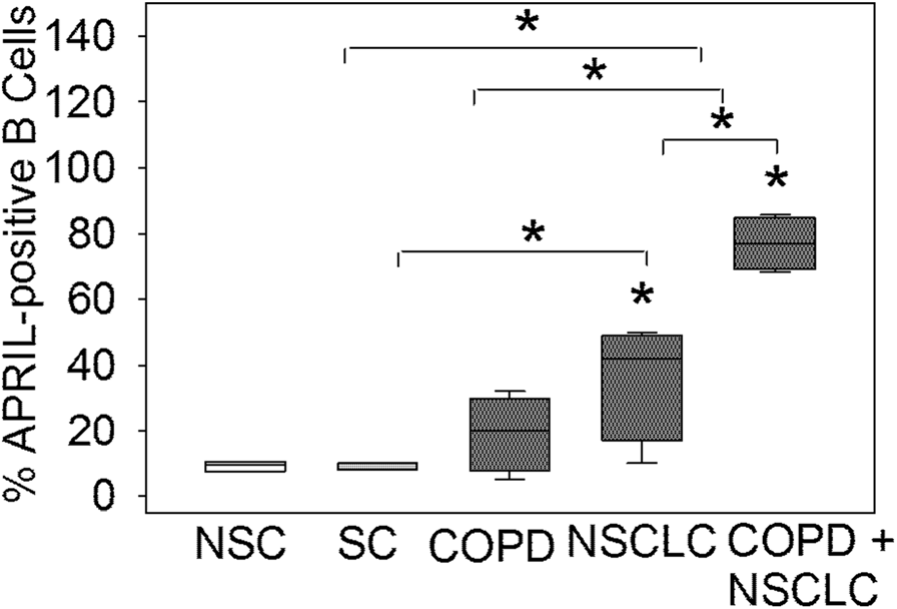
The proportion of APRIL-expressing B cells in the lungs of control subjects, and patients with COPD alone, NSCLC alone or patients with both COPD and NSCLCs: The percentages of APRIL-positive B cells in the lungs of NSC, SC, patients with COPD alone, NSCLC alone and both COPD and NSCLC are shown. The box plots show the median values and the 5 and 95 % confidence intervals, and the error bars are the standard deviations. * indicates *p* < 0.03 vs. NSC or vs. the group indicated

All studies conducted on human subjects were approved by the Partners Institutional Review Board. The characteristics of the lung tissue cohort studied are shown in Fig. 1. De-identified sections of lung were obtained from lung biopsies, or from lung explants from COPD patients provided by NHLBI-sponsored Lung Tissue Research Consortium (www.ltrcpublic.com), or the Department of Pathology at Brigham and Women’s Hospital, Boston. None of the subjects studied had evidence of respiratory tract infection at the time of lung tissue sampling. Among the subjects studied, 8 subjects had a primary diagnosis of COPD (forced expiratory volume in 1 s/forced vital capacity [FEV_1_/FVC] < 0.7), 4 subjects had a primary diagnosis of NSCLC, 3 were healthy ever-smokers (SC), and 3 were healthy never-smokers (NSC). Among the COPD patients, 4 had a secondary diagnosis of NSCLC.

#### Immunostaining of lung sections for APRIL

Formalin-fixed and paraffin-embedded sections (5 μm thick) of peripheral lung sections from the subjects were studied. Lung sections were deparaffinized, and antigen (Ag) retrieval was performed by boiling the slides immersed in 0.01 M sodium citrate and 2 mM citrate buffer (pH 6.0) in a microwave. We used a rabbit anti-APRIL IgG followed by goat anti-rabbit F(ab’)_2_ conjugated to Alexa-488 to identify APRIL-positive cells. PMNs were identified by their characteristic multi-lobed nuclei (identified by counter-staining lung sections with 4′,6-diamidino-2-phenylindole [DAPI]). AMs were defined as mononuclear cells (having ample cytoplasm) present in the alveolar spaces. Bronchial and alveolar epithelial cells were identified in bright-field images. To identify B cells, we immunostained the sections with a murine anti-CD20 IgG followed by a goat anti-murine IgG conjugated to Alexa-546. All of the antibodies (Abs) listed were purchased from Abcam (Cambridge, MA) and secondary antibodies were obtained from ThermoFisher Scientific (Cambridge, MA). Lung sections were also immuno-stained with appropriate isotype-matched non-immune control antibodies. Images of the immuno-stained lung sections were captured and analyzed using an epi-fluorescence microscope (Leica Microsystems, Buffalo Grove, IL).

In order to quantify APRIL expression in PMNs and B cells, PMNs and B cells in at least 50 non-consecutive high-magnification fields were analyzed. At least 20 AMs within the alveolar spaces were evaluated for each patient. Results were expressed as percentage of all AMs, PMNs and B-cells that were counted that stained positively for APRIL. For evaluation of APRIL in AECs, cells in the alveolar walls in 10 non-consecutive high magnification fields were evaluated for each subject. The area of the alveolar wall was measured using MetaMorph software (Molecular Devices, Sunnyvale, CA). APRIL-positive cells within the alveolar walls were counted and the results were expressed as the number of positive cells/pixel of alveolar wall.

#### Statistical analyses

Data were analyzed using one-way ANOVA tests for continuous variables (age, FEV_1_ % predicted and pack/years) followed by pair-wise comparisons using student’s t-tests or Mann-Whitney U tests. The Chi-square test was used to analyze categorical variables. *P* < 0.05 was considered statistically significant.

## Results

### Demographics

The demographic characteristics of the patients are shown in Table 1. NSC, SC, COPD patients and patients with cancer did not differ in their sex ratios or pack-year smoking histories. The age of the patients having both COPD and NSCLC was significantly higher than that of the patients with COPD alone. The smoking pack-year histories of the SC, patients with COPD alone or NSCLC alone were similar, and all were significantly higher than that of the NSC by design. The pack-year smoking histories were not available for patients with COPD and NSCLC. The FEV_1_ as a percentage of predicted (FEV_1_ % pred) was not available for the NSC and was only available for one of the four patients with both COPD and NSCLC. In the latter group, COPD was diagnosed on the basis of the presence of emphysema in high resolution computed tomography (HRCT) scans of the thorax and an FEV_1_/FVC ratio < 0.7, but the FEV_1_% predicted was not available from the electronic medical records. There was a significant difference in FEV_1_ % pred between COPD patients and both SC and NSC by design.

**Table 1:** The table shows the demographic and clinical characteristics of the healthy never smokers (NSC), healthy smokers (SC) and patients with COPD alone, NSCLC alone and both COPD and NSCLC. SC were defined as subjects that were current smokers at the time of the study or had quit smoking less than 1 year before the lung samples were obtained.

Demographics and clinical characteristics of the subjects						
Characteristics	NSC (*n* = 3)	SC (*n* = 3)	COPD^a^ (*n* = 3)	COPD + Cancer (*n* = 3)	Cancer (*n* = 3)	*P* ^e^
% males	33	33	75	75	0	N.S.
Age (yrs)	65 ± 22	63 ± 15	56 ± 7	75 ± 12	64 ± 9	*P* = 0.03^b^
Pack-yr smoking^c^	0	33 ± 23	45 ± 26	N/A	50 ± 10	*P* < 0.001
FEV_1_ (% of predicted)^d^	N/A	87 ± 11	33 ± 20	61^f^	61 ± 7	*P* < 0.05

^a^ All COPD patients had forced expiratory volume in 1 s/forced vital capacity (FEV_1_/FVC) < 0.7 whereas smokers without COPD and non-smoker controls had FEV_1_/FCV > 0.7

^b^ The results for age, pack-year smoking history and FEV_1_ % predicted, are expressed as mean ± SEM. The age of the patients with both COPD and NSCLC was significantly higher than that of the patients with COPD alone

^c^ The pack-year smoking histories of the COPD patients and SC groups were significantly different from those of NSC by design (*P* < 0.001 for both comparisons). The pack-yr smoking histories of the patients with COPD alone and NSCLC alone were significantly different from those of the SC and NSC

^d^ The FEV_1_ % predicted and the FEV_1_/FVC (not shown) in the COPD group were significantly different from that of the SC and NSC by design (*P* < 0.05 for both comparisons)

^e^ Statistical analyses included one-way ANOVA tests for continuous variables (age, FEV_1_ % predicted and pack/years) followed by pair-wise comparisons using student’s t-tests or Mann-Whitney U tests. The Chi-square test was used to analyze categorical variables. *P* < 0.05 was considered statistically significant

^f^ The FEV_1_ % predicted in the group of subjects with COPD and NSCLC was available only in 1 out of 4 subjects

### The proportion of APRIL-expressing AMs and B cells is higher in the lungs of patients with both COPD and NSCLC versus COPD alone, NSCLC alone or control subjects

APRIL expression was detected in some B cells, AECs and AMs in lung sections from all subjects studied albeit at low levels in SC and NSC. No staining was present in bronchial epithelial cells in any of the subject groups, or in lung sections stained with non-immune isotype-matched non-immune antibodies.

The percentage of APRIL-positive B cells was higher in the lung sections from patients with both COPD and NSCLC when compared to patients with COPD alone, NSCLC alone, SC and NSC (Fig. 1; *p* < 0.005 for all comparisons). The percentage of APRIL-positive B cells was higher in lung sections from patients with NSCLC alone than in the lungs of SC or NSC (*p* < 0.03 for both comparisons; Fig. 1). The percentage of APRIL-positive B cells was similar in lung sections from patients with NSCLC alone versus COPD alone; patients with COPD alone versus SC and NSC; and also SC versus NSC.

### The number of APRIL-expressing AECs is higher in patients with both COPD and NSCLC versus COPD alone, NSCLC alone or control subjects

**Fig. 2:**
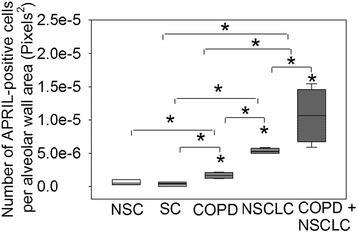
The proportion of APRIL-expressing AECs in the alveolar walls of control subjects, and patients with COPD alone, NSCLC alone or patients with both COPD and NSCLCs: The percentages of APRIL-positive AECs in the alveolar walls of NSC, SC, patients with COPD alone, NSCLC alone and both COPD and NSCLC are shown. The box plots show the median values and the 5 and 95 % confidence intervals, and the error bars are the standard deviations. * indicates *p* < 0.03 vs. NSC or vs. the group indicated

Patients with both COPD and NSCLC had a higher number of APRIL-positive AECs per area of alveolar wall than patients with either COPD alone or NSCLC alone (*p* < 0.03 for both comparisons; Fig. 2) or SC or NSC (*p* < 0.005 for both comparisons; Fig. 2). The number of APRIL-positive AECs was higher in the alveolar walls of patients with either COPD alone or NSCLC alone than in the alveolar walls of SC and NSC (*p* < 0.02 for all comparisons; Fig. 2). The numbers of APRIL-positive AECs were similar in the alveolar walls of SC and NSC. No staining for APRIL was detected in bronchial epithelial cells in lung sections from any of the subject group studied (data not shown).

### The proportion of APRIL-expressing AMs is higher in patients with both COPD and NSCLC versus COPD alone, NSCLC alone or control subjects

**Fig. 3:**
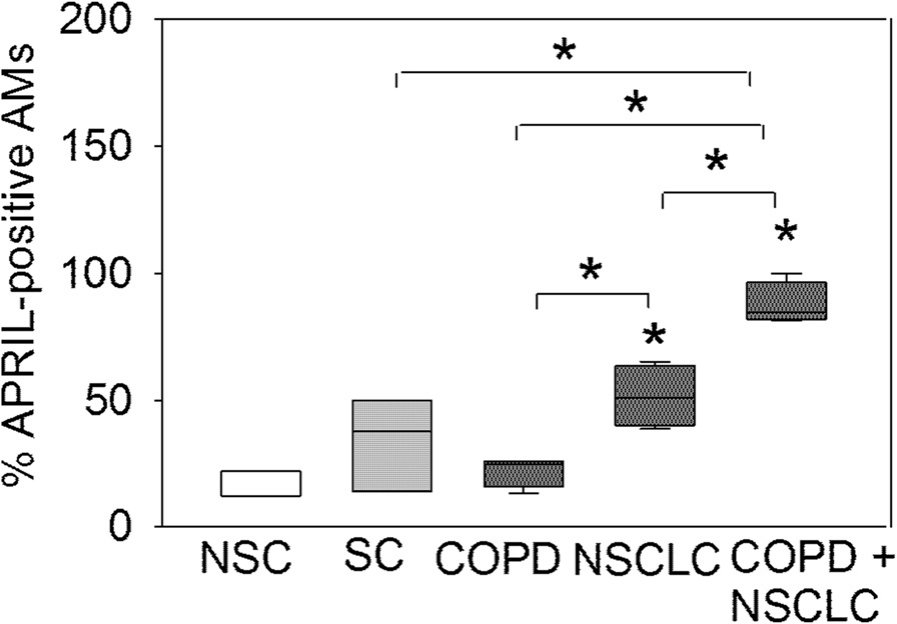
The proportion of APRIL-expressing AMs in the lungs of control subjects, and patients with COPD alone, NSCLC alone or patients with both COPD and NSCLC: The percentages of APRIL-positive AMs in the lungs of NSC, SC, patients with COPD alone, NSCLC alone and both COPD and NSCLC are shown. The median and 5 and 95 % confidence intervals are shown in the box plots and the error bars in the box plots are standard deviations. * indicates *p* < 0.03 vs. NSC or vs. the group indicated

The percentage of APRIL-positive AMs was higher in lung sections from patients with both COPD and NSCLC versus NSCLC alone, COPD alone, SC or NSC (Fig. 3, *p* < 0.003 for all comparisons). The percentage of APRIL-positive AMs was higher in patients with NSCLC alone than in patients with COPD alone or NSC (*p* < 0.03 for both comparisons; Fig. 3). The percentage of APRIL-positive AMs was similar in lung sections from patients with COPD and SC, and also in SC and NSC.

### The proportion of APRIL-expressing PMNs is higher in patients with COPD and NSCLC versus COPD alone, or NSCLC alone

**Fig. 4:**
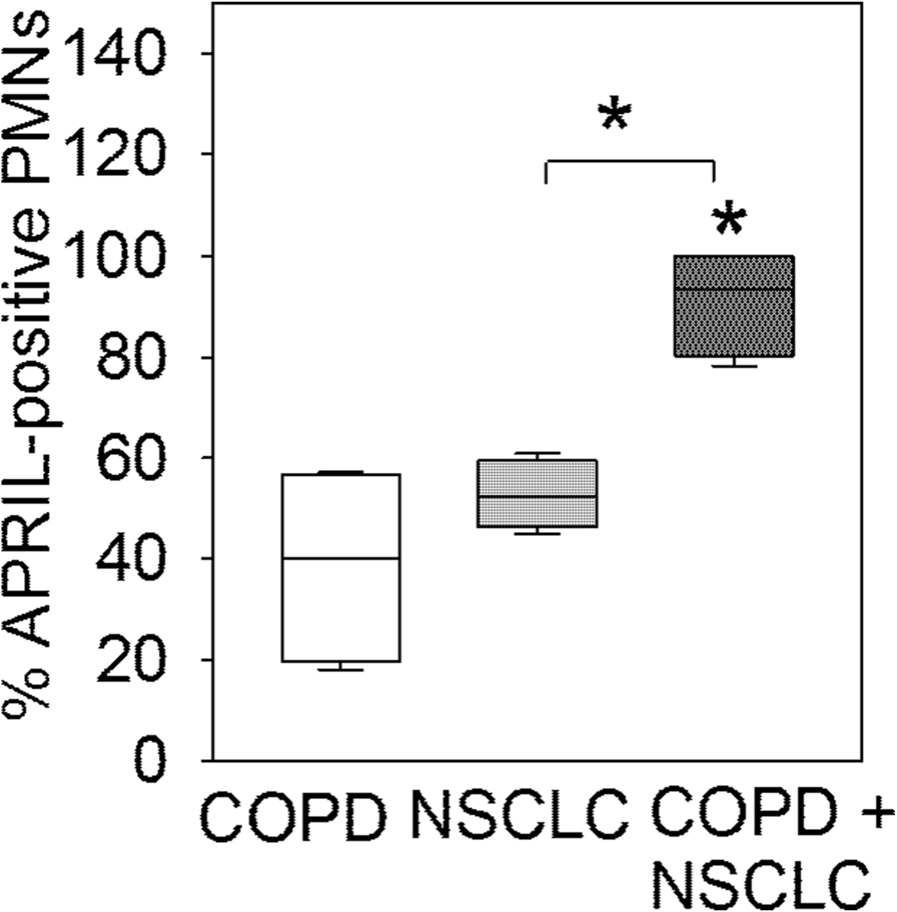
The proportion of APRIL-expressing PMNs in the lungs of control subjects, and patients with COPD alone, NSCLC alone or patients with both COPD and NSCLC: The percentages of APRIL-positive PMNs in the lungs of NSC, SC, patients with COPD alone, NSCLC alone and both COPD and NSCLC are shown. The box plots show the median values and the 5 and 95 % confidence intervals, and the error bars are the standard deviations. * indicates *p* < 0.03 vs. NSC or vs. the group indicated

The percentage of APRIL-positive PMNs was higher in lung sections from patients with COPD and NSCLC than in patients with either COPD alone or NSCLC alone (Fig. 4, *p* < 0.03 for both comparisons). Although low-level positive staining for APRIL was detected in PMNs in lung sections from SC and NSC, we could not quantify the expression of APRIL in cells from these subjects due to the very small number of PMNs present in peripheral lung sections from these subject groups.

## Discussion

We report for the first time that there are increases in numbers of APRIL-expressing leukocytes and AECs in lungs of patients with either COPD or NSCLC versus control subjects without these diseases. APRIL-expressing cells in COPD and NSCLC lungs include B cells, AMs, PMNs and AECs but not bronchial epithelial cells. Patients with both COPD and NSCLC had a higher percentage of APRIL-expressing leukocytes and AECs than patients with either disease alone. We hypothesize that increased APRIL expression in leukocytes recruited to the lung or in AECs is linked to the progression of both COPD and NSCLC, and possibly also to the development and progression of NSCLC in patients with established COPD.

### APRIL expression in COPD and NSCLC

To our knowledge, APRIL has never been studied in the lungs of COPD patients. Prior studies of NSCLC report that APRIL expression is increased in tumor cells and stromal cells in the vicinity of the tumors [30]. The activities of APRIL in regulating NSCLC development and progression are unclear as APRIL promotes the proliferation of several tumor cell lines in vitro and *in vivo* [31] but promotes apoptosis of other tumor cell types [32, 33]. Interestingly, APRIL expression levels in NSCLC cells are an independent prognostic factor for 5-year survival in NSCLC patients [30], suggesting that APRIL expression promotes the progression of NSCLC tumors. Whether APRIL expression in leukocytes and/or AECs can serve as a prognostic marker for either COPD or NSCLC was not studied herein, but will be the focus of our future studies.

### Leukocyte-derived APRIL in COPD and NSCLC

We detected impressive increases in APRIL expression in B cells, AMs and PMNs in the lungs of patients with either COPD or NSCLC and even greater increases in APRIL expression in these leukocytes in the lungs of patients with both diseases. APRIL is produced by a variety of cell types such as macrophages, monocytes, dendritic cells and T lymphocytes [28, 34]. APRIL secreted by these cells plays a key role in peripheral B cell survival, maturation and differentiation [28]. B cells have been strongly linked to COPD pathogenesis by producing auto-antibodies that promote an inflammatory response that injures the alveolar walls [12, 13, 25, 35]. A high density of follicular B cells correlates with long-term survival in patients with NSCLC treated with chemotherapy [17]. However, IL-10-producing immunosuppressive regulatory B cells are also increased in NSCLC, and their numbers correlate directly with disease progression [18]. We did not determine whether APRIL is expressed in different B cell subsets in the lungs in our study. However, future studies will determine whether APRIL expression is increased mainly in regulatory B cells and promotes the survival of this subset to drive NSCLC progression.

The activity of APRIL in regulating the functions of leukocytes other than B cells that have been strongly implicated in the pathogenesis of COPD and NSCLC has been much less well studied. APRIL is expressed by peripheral blood PMNs from healthy subjects, and APRIL expression in blood PMNs is increased in patients with B cell lymphomas and associated with reduced expression of the apoptosis-inducing ligand TRAIL [36]. However, the expression of APRIL in blood or lung PMNs in patients with COPD and NSCLC has not been examined previously. Whether the increased APRIL expression in PMNs in patients with COPD and NSCLC leads to alterations in PMN function (including apoptosis or survival) that promote disease progression in COPD or NSCLC will be examined in future studies.

Macrophages from tissues other than the lung are known to express APRIL when activated with tumor growth factor beta-β, interleukin 4 and other mediators in vitro [37, 38]. However, to our knowledge APRIL has not been reported to be expressed by AMs previously. We detected low-level APRIL expression in AMs in the lungs of both NSC and NSC. A greater proportion of AMs from patients with either COPD or NSCLC expressed APRIL than cells in control subjects, and an even greater proportion of patients with both diseases expressed this molecule. APRIL induces activation of macrophage-like cells lines by activating NF-kB [39] which is an important pro-inflammatory transcription factor in COPD lungs [40]. Likely, the increased APRIL expression by macrophages that we detected contributes to macrophage activation and macrophage-mediated pulmonary injury in COPD lungs. Interestingly, activation of NF-kB in myeloid cells is crucial for promoting growth of lung cancer cells in murine models [41]. Whether macrophage APRIL expression is linked to disease progression in either COPD or NSCLC is not clear but will be the focus of our future studies.

### APRIL expression by AECs in COPD and NSCLC

Normal bronchial epithelial cells and BEAS2B cell lines express APRIL at low levels in vitro and expression increases when cells are incubated with double stranded RNA [24], but APRIL expression by AECs has not been reported previously. Surprisingly, our study reports that AECs (but not bronchial epithelial cells) in normal lungs have detectable (albeit low-level) APRIL expression. A greater proportion of AECs (but not bronchial epithelial cells) in patients with either COPD or NSCLC patients express APRIL, and patients with both diseases had the highest proportion of APRIL-expressing AECs. APRIL is expressed in epithelial cells in other organs and may contribute to pathologies occurring in other diseases. For example, human intestinal epithelial cells trigger IgA(2) class switching in B cells recruited to mucosal follicles by releasing APRIL after sensing bacteria through Toll-like receptors [42]. Thus, it is possible that APRIL expressed by AECs in the lungs of patients with COPD and NSCLC contributes to the progression of each disease by activating B cells and macrophages in the lungs (and/or tumor cells in the case of patients with NSCLC). Whether APRIL regulates the activities of AECs (such as maintenance of the lung structure, barrier function and secretion of pro-inflammatory or pro-angiogenic cytokines, growth factors and host defense proteins) [43–47] that could contribute to the progression of COPD or NSCLC is not clear.

### Linking APRIL to the development of NSCLC in COPD patients

Herein, we report for the first time that APRIL expression was significantly higher in lung leukocytes and AECs in patients having both COPD and NSCLC, than in patients with either COPD alone or NSCLC alone. A common link between COPD and NSCLC is the presence of chronic pulmonary inflammation consisting of cells of both innate and adaptive immune systems, as aberrant inflammation has been proposed as the common link between these two pathologies [48]. There is substantial evidence that chronic pulmonary inflammation promotes the development and progression of COPD [25, 49, 50]. In NSCLC, there is a marked infiltration of cells of both innate and adaptive immune system, and the cell types and their tissue localization are significantly associated with progression and survival. As is the case in COPD patients, NSCLC patients have tertiary lymphoid structures (TLS) characterized by clusters of mature dendritic cells and T cells surrounded by B cells [13, 17]. Leukocytes have both tumor-promoting and -inhibiting roles [51, 52]. Although we detected increases in APRIL expression in PMNs and AMs in patients with NSCLC, the activities of APRIL in regulating PMN and AM function (such as their activation states, survival and ability to removed transformed cells) were not examined in our study, but will be the focus of future studies. However, our results raise the intriguing possibility that increased APRIL expression in lung leukocytes in COPD patients further activates these cells to produce more APRIL. This, in turn, could create an environment conducive to tumorigenesis as reactive oxygen species released by leukocytes promote formation of reactive carbonyls that are not only tumorigenic by initiating DNA damage [53], but can directly alter the function of regulatory proteins involved in host immunity and having tumor suppressor functions [54]. Leukocytes also release various growth factors and pro-angiogenic mediators that promote tumor development [55]. Alternatively, the increased APRIL expression by AECs in COPD lungs could promote tumorigenesis by inducing excessive proliferation of bronchial epithelial cells. Once NSLC is established in COPD lungs, the high levels of APRIL produced by leukocytes, AECs, and/or tumor cells themselves could promote NSCLC progression as APRIL potently promotes survival of tumor cells in vitro, and also when human tumors are transplanted into immune-deficient mice [31].

### Limitations of our study

Our study has several limitations. First, we studied a small number of subjects. Patients with both COPD and lung cancer were significantly older than those with COPD alone. Second, in the group of patients with both COPD and NSCLC, only one out of four patients had PFT values available, and the pack-year smoking histories were not available for this group. In the latter group COPD was diagnosed using HRCT scans and FEV_1_/FVC ratios < 0.7 (but the absolute FEV_1_ and FVC values were not reported). Thus, we were not able to correlate APRIL expression with COPD severity, pack-year smoking history, current versus former smoker status or NSCLC cell type. Another limitation of our study is that we did not measure the APRIL expression levels per cell (only the percentage of APRIL-expressing cells). We also did not correlate APRIL expression with readouts of cellular functions germane to COPD or NSCLC. These studies will be the focus of future studies in our laboratory.

## Conclusions

Herein, we report an increased APRIL expression in lung leukocytes and AECs in patients with COPD alone and NSCLC alone, and even greater increases in these cells in patients with both diseases. Our results suggest that exaggerated APRIL expression in lung leukocytes and/or AECs could create an environment conducive to the development and/or progression of NSCLC in patients with COPD.
